# Interactions of socioeconomic position with psychosocial and environmental correlates of children's physical activity: an observational study of South Australian families

**DOI:** 10.1186/1479-5868-6-56

**Published:** 2009-08-14

**Authors:** James Dollman, Nicole R Lewis

**Affiliations:** 1 Nutritional Physiology Research Centre, University of South Australia, Adelaide, South Australia; 2 School of Health Sciences, University of South Australia, Adelaide, South Australia

## Abstract

**Background:**

Evidence for psychosocial and environmental correlates on children's physical activity is scattered and somewhat unconvincing. Further, the moderating influences of socioeconomic position (SEP) on these influences are largely unexplored. The aim of this study was to examine the interactions of SEP, operationalised by mother education, and predictors of children's physical activity based on the Youth Physical Activity Promotion Model.

**Methods:**

In 2005, a sample of South Australians (10–15 y) was surveyed on psychosocial and environmental correlates of physical activity using the Children's Physical Activity Correlates Questionnaire (n = 3300) and a parent survey (n = 1720). The following constructs were derived: 'is it worth it?' (perceived outcomes); 'am I able?' (perceived competency); 'reinforcing' (parental support); and 'enabling' (parent-perceived barriers). Self-reported physical activity was represented by a global score derived from the Physical Activity Questionnaire for Adolescents. Associations among physical activity and hypothesised correlates were tested among children with mothers of high (university educated) and low (left school at or before 15 y) SEP.

**Results:**

Among high SEP children, 'is it worth it?' emerged as a significant predictor of physical activity for boys and girls. Among low SEP children, 'is it worth it?' predicted boys' physical activity, while among girls, 'reinforcing' was the only significant predictor, explaining ~35% of the total explained variance in physical activity.

**Conclusion:**

While perceived outcomes emerged as a consistent predictor of physical activity in this sample, parental support was a powerful limiting factor among low SEP girls. Interventions among this high risk group should focus on supporting parents to provide both emotional and instrumental support for their daughters to engage in physical activity.

## Background

Physical inactivity and high sedentariness have been associated with negative health outcomes for both adults [1] and children [2]. There is widespread evidence for poorer health among adults [3] and children [4-6] of low socioeconomic position (SEP), across a range of health indicators. Gradients in physical activity behaviours that parallel SEP gradients in health have been reported among adults [7] and children [8-12]. A recent study [10] identified inverse associations among SEP and screen-based leisure time in South Australian youth, while the same trend has also been reported from various European countries [11] and the United States [8]. Furthermore, there have been more marked declines in active transport between school and home, school sport, and physical education participation among low SEP compared with high SEP children in Victoria, Australia, between 1985 and 2001 [12].

There is a broad range of social, psychological and environmental factors identified as correlates of youth physical activity [13], and several theoretical models proposed to explain variance in physical activity behaviour. The Youth Physical Activity Promotion Model (YPAP) has recently been offered as an ecological framework for understanding the inter-connectedness of these influences on youth physical activity, by merging several theories into one [14]. Components of the model include psychological attributes ('predisposing factors'), social influences ('reinforcing factors'), and environmental influences ('enablers').

Despite the growing determinants literature, our understanding of influences on youth physical activity remains clouded. There are very few factors that consistently predict youth physical activity across the majority of studies, prompting the authors of the classic review of this area [13] to call for predictors to be studied in more clearly defined socio-demographic groups. The development of intervention strategies has been primarily based on cross-sectional observations of correlates of healthy behaviours in heterogeneous samples, and has accordingly followed a 'one size fits all' approach, with largely disappointing outcomes [15]. The capacity to address socioeconomic disparities in youth physical activity and associated health outcomes will depend on understanding how correlates of physical activity function differentially across SEP strata. For instance, among low SEP children it is reasonable to postulate that stronger parental support is required to overcome the barriers associated with economic disadvantage, such as access to facilities and costs of participation [15,16].

The aim of this study was to examine the interactions of SEP, operationalised by mother education, and correlates of youth physical activity based on the YPAP model [14]. Specifically, it is hypothesised that relationships between psychosocial and environmental factors and children's physical activity are moderated by maternal education.

## Methods

### Participants

In 2005, a sample of South Australians (10–15 y) was assessed through student (n = 3300) and parent (n = 1720) surveys. To ensure representation across the socioeconomic spectrum, a listing was obtained of all South Australian government and independent schools, along with the School Card Register (SCR; the percentage of students enrolled at the school receiving government support for low-income families). A high SCR score denotes a higher percentage of children receiving government assistance, and thereby is an inverse of SEP at the school level. Four quartile bands were identified according to SCR (0–19, 20–39, 40–59, and ≥ 60%), with the selection of schools from each band proportional to the number of schools per band. Within schools, all children in grades 5 to 10 were invited to participate. Remote and special schools were excluded from the sample due to low student enrolments and issues with self-report in these populations, respectively. Of 70 schools invited, 52 (74.3%) agreed to participate. From these participating schools, 5348 children were eligible, 3754 (70.2%) gave consent to participate and 3300 provided complete survey responses. Representation of schools in each quartile band was 26% (0–19 band), 49% (20–39 band), 19% (40–59 band) and 7% (60+ band), compared with the state-wide percentage of schools in each quartile band (19%, 46%, 24% and 10% respectively).

### Measurement instruments

Two questionnaires collected information on family demographics, child physical activity and its hypothesised predictors. The children's questionnaire was completed in the classroom setting, administered by teachers according to a standardised script. Children took home a questionnaire for parents/caregivers to complete and return to the school.

### The Children's Questionnaire

Two sections comprised the Children's Questionnaire. A psychosocial questionnaire, the Children's Physical Activity Correlates (CPAC), includes 44 items that assess various psychosocial correlates of physical activity. The instrument includes 15 items from the Children's Attraction to Physical Activity scale, five items from Harter's perceived competence scale, 6 items from Rosenberg's self-esteem scale and 18 items from a parent socialisation scale [17]. In the YPAP model, Welk [14] has postulated domains derived from the CPAC: parental influence ('reinforcing'); and predisposing factors [attitudes to physical activity ('is it worth it?') and perceived competence ('am I able?')]. In the current study, internal consistency for these three scales was acceptable: 'reinforcing', α = 0.74; 'is it worth it?', α = 0.84; and 'am I able?', α = 0.77).

The Physical Activity Questionnaire for Adolescents (PAQ-A) asks respondents to recall the number of times they performed moderate to vigorous physical activity in the previous week, choosing from a checklist. Seven questions assess physical activity in both school- and out-of-school-hours, covering physical education, lunch, after school, evenings, and the weekend. The items of the PAQ-A are scored on a 5-point scale and a composite index (global PA) is formed as the average of these scores. The questionnaire has exhibited acceptable validity and reliability in previous studies [18-20].

### The Parent Questionnaire

The parent survey assessed physical environmental factors, focusing on: risks to safety (strangers and traffic); access to facilities and play opportunities (playgrounds and other children in the neighbourhood); and transport availability. These constructs represented the 'enabling' domain of the YPAP model [14]. The questionnaire was adapted from a study by Sallis and colleagues [21], who reported test-retest reliabilities 0.68–0.89 for neighbourhood characteristics and access to facilities among US college students. Additional questions represented parents' leisure-time physical activity, based on the Transtheoretical Model of Change [22]. Individual items from the Parent Questionnaire are presented in Table 1.

**Table 1:** Items from the parent survey (and response options) for enabling factors and parent physical activity

Variable	Items	Response options (data code in brackets)
neighbourhood risk (mean of 3 items)	It is not safe for my child to play, walk or ride a bike near the house because of traffic/strangers (2 items)	strongly disagree (1); disagree (2); unsure (3); agree (4); strongly agree (5)
	My child is allowed to play outside of my property alone, or with friends, without adult supervision*	strongly disagree (1); disagree (2); unsure (3); agree (4); strongly agree (5)

access (mean of 3 items)	There are parks, recreation facilities and other play areas within my child's walking distance from home*	strongly disagree (1); disagree (2); unsure (3); agree (4); strongly agree (5)
	It is easy to arrange transport for my child to get to sporting activities*	strongly disagree (1); disagree (2); unsure (3); agree (4); strongly agree (5)
	There are not enough children of similar age near where we live, for my/our child to play with	strongly disagree (1); disagree (2); unsure (3); agree (4); strongly agree (5)

mother/father activity (mean of 2 items)	Which one of the following best characterises your activity habits? Physically active implies that you get at least 30 minutes of moderate physical activity on most days of the week or 20 minutes of vigorous activity on at least 3 days a week.	I am not physically active and I don't plan to start (1); I am not physically active, but I intend to start (2); I am occasionally active, but not regularly (3); I have been physically active recently, but for less than 6 months (4); I have been physically active regularly for the past 6 months (5)

Note:

* Denotes survey item was reverse coded

### Parent education

The parent questionnaire included items on mother's and father's education level, according to the following classifications: still at school (coded 1); left school at 15 years or less (2); left school after age 15 (3); left school after age 15 but still studying (4); trade/Apprenticeship (5); post-secondary certificate/diploma (6); university degree or higher (7). In this analysis, mother education was used to represent SEP, as this attribute is consistently associated with youth overweight and obesity [4], and with adolescent physical activity [23].

### Statistical Analysis

Scores for each YPAP construct were derived by averaging individual item responses (coded 1 to 4). For each construct, higher scores represented higher predisposition ('is it worth it?' and 'am I able?'), parental support ('reinforcing') and environmental support ('enabling'). Descriptive statistics (sex-specific means +/- SD) for global PA and all predictors were calculated, and comparisons between boys and girls performed using ANCOVA, adjusting for age.

Stepwise multiple regression models of global PA and predictors based on the YPAP model were established, controlling for age. Because children were sampled in schools, regression modelling was conducted with robust standard errors to account for clustering of measured attributes among children in the same school. These models were tested separately in boys and girls, and included interaction terms of mother education and each of the YPAP variables. As some interaction terms were significant, regression models of global PA and predictors, controlling for age and accounting for design effects, were repeated in separate subsamples based on mother education: low, mother education ≤ 2 (did not complete secondary education); and high, mother education = 7 (completed a University degree). This stratification was confined to high and low mother education categories to maintain a clear distinction between strata; mother education levels 3 to 6 is quite a 'heterogeneous' group, including those who left school before completing the final secondary year, those still pursuing secondary qualifications, and those with post-secondary diplomas and certificates.

Statistical significance was inferred at p ≤ 0.05. All statistical procedures were conducted using STATA [version 9] (Stata Corporation, College Station, USA, 2003).

## Results

Among subjects who completed the student survey, those who did, and did not, return a completed parent survey were compared on parent education, age and sex. Not surprisingly, those who did provide parent data were younger (11.7 vs 13.3 y). There were no differences by sex or parent education.

Compared with girls, boys reported higher levels of global PA and more positive influences on physical activity, with the exceptions of mother's and father's physical activity (see Table 2). From regression models of global PA and YPAP constructs in the whole sample, 'reinforcing' and 'is it worth it?' emerged as significant predictors, among boys and girls (see Table 3). Among girls, interactions of constructs with mother education were also significant. The correlation of 'reinforcing' with global PA was weaker among girls with university-educated mothers, while the correlation of 'is it worth it?' was stronger among girls with university-educated mothers. For boys, 'am I able' and its interaction with mother education were significant predictors of global PA. In all regression models, age emerged as a significant predictor of global PA, with higher levels among younger children.

**Table 2:** Comparisons between males and females on global physical activity and its hypothesised predictors

	boys	girls	*p *for comparison
Age	12.03 (1.45)	11.97 (1.41)	0.43
Global PA	3.00 (0.71)	2.72 (0.65)	<0.0001
Is it worth it?	3.29 (0.49)	3.13 (0.49)	<0.0001
Am I able?	3.04 (0.44)	2.86 (0.49)	<0.0001
Reinforcing	3.09 (0.46)	3.05 (0.46)	0.05
Enabling	3.53 (0.68)	3.40 (0.71)	<0.0001
Father PA	3.80 (1.11)	3.75 (1.19)	0.30
Mother PA	3.75 (1.24)	3.70 (1.26)	0.38

Note:

PA = physical activity

**Table 3:** Predictors of global physical activity in the whole sample

Predictor	Coefficient	SE	Beta	*p*	Partial R^2^
	*Boys (n = 691)*				
Age	-0.11	0.01	0.22	<0.0001	0.05
Is it worth it?	0.62	0.06	0.43	<0.0001	0.26
Reinforcing	0.22	0.06	0.14	<0.0001	0.02
Enabling	0.07	0.03	0.07	0.004	0.01
Mother education* enabling	-0.007	0.002	-0.07	0.01	0.01
	*Model: F(5,47) = 87.45, R*^2 ^= *0.35, p < 0.0001*				

	*Girls (n = 827)*				

Age	-0.11	0.02	-0.24	<0.0001	0.05
Is it worth it?	0.26	0.10	0.19	0.01	0.01
Reinforcing	0.48	0.10	0.32	<0.0001	0.19
Mother education* is it worth it?	0.05	0.02	0.49	0.007	0.02
Mother education* reinforcing	-0.06	0.02	-0.51	0.005	0.02
	*Model: F(5,48) = 37.04, R*^2 ^= *0.29, p < 0.0001*				

Different patterns of predictors emerged in sub-samples formed on the basis of mother education, particularly for girls (see Tables 4, 5). For children with University educated mothers, 'is it worth it?' emerged as a significant predictor, while for boys in this group, father activity was also a significant but relatively weak predictor of global PA. Among those with poorly educated mothers, 'is it worth it?' predicted boys' global PA, but among girls 'reinforcing' was the only significant predictor, explaining 35% of the total explained variance in global PA (see Table 5).

**Table 4:** Predictors of global physical activity among children with University educated mothers

Predictor	Coefficient	SE	z	p	*Partial R*^2^
	*Boys (n = 130)*				
Age	-0.10	0.02	-0.21	<0.0001	*0.04*
Is it worth it?	0.80	0.14	0.57	<0.0001	*0.38*
Father PA	0.07	0.03	0.11	0.04	*0.01*
	*Model: F(3,32) = 16.76, R*^2 ^= *0.43 p < 0.0001*				

	*Girls (n = 130)*				

Age	-0.15	0.03	-0.30	<0.0001	*0.08*
Is it worth it?	0.74	0.09	0.53	<0.0001	*0.27*
	*Model: F(2,30) = 34.20, R*^2 ^= *0.36, p < 0.0001*				

**Table 5:** Predictors of global physical activity among children with mothers who did not complete secondary education

Predictor	Coefficient	SE	Beta	p	*Partial R*^2^
	*Boys (n = 40)*				
Age	-0.17	0.06	-0.34	0.007	*0.09*
Is it worth it?	0.72	0.27	0.40	0.02	*0.16*
	*Model: F(2,22) = 8.39, R*^2 ^= *0.26, p = 0.002*				

	*Girls (n = 59)*				

Age	-0.14	0.04	-0.28	0.001	*0.08*
Reinforcing	0.65	0.15	0.51	<0.0001	*0.35*
	*Model: F(2,26) = 18.40, R*^2 ^= *0.44, p < 0.0001*				

Similar patterns of predictors emerged in models for sub-groups formed on the basis of father education; only those based on mother education are reported here.

## Discussion

This study examined correlates of physical activity among children from contrasting SEP, as defined by mother education. Consistent with the published literature, the results identified higher levels of physical activity among boys compared with girls, and younger compared with older children [13]. Among low SEP boys and high SEP boys and girls, physical activity was associated with perceptions of outcomes ('is it worth it?'). These findings resonate with a national survey of USA pre-adolescents (9–13 y) that identified outcomes expectations as the only psychosocial predictor of both non-organised and organised physical [24].

The findings of the current study extend the literature by identifying a moderating effect of mother's education on the association of parental influences and girls' physical activity. Specifically, parental support ('reinforcing') was a limiting factor for physical activity among low, but not high, SEP girls. This suggests that parental support plays an important role in assisting girls to overcome barriers to physical activity opportunities that are more potent in low SEP communities [16]. Further, low SEP girls with unsupportive parents appear to be at greatest risk of low habitual physical activity. On the other hand, high SEP girls might be less dependent on supportive parents if surrounded by more abundant opportunities and fewer restrictions to be physically active. It is unclear why parental support was a limiting factor for physical activity among low SEP girls, but not low SEP boys. Higher physical activity levels among boys, consistently reported in the literature [13], may reflect a higher internal drive among boys that predisposes them to greater participation regardless of influences from their social and physical environments.

Family support is a widely reported predictor of physical activity among young females [25,26]. Notably, declines in perceived social support have been reported in girls followed between the 5^th ^and 7^th ^grades [27]. This mirrors the widely reported decline in girls' physical activity through adolescence [28]. Kimm and colleagues [29] reported declines in North American females' physical activity of 64% in White girls and 100% in Black girls over a ten year period. In the same study, girls reporting higher perceived family support in the 8th grade were more physically active in 12^th ^grade, independent of their self-efficacy or perceived behavioural control, suggesting that higher family support may attenuate the age-related decline in adolescent girls' physical activity. Interventions focusing on factors that are most proximal to the targeted behaviour have a greater likelihood of success [16]. The current study underscores the critical role played by parents in helping low SEP girls maintain active lifestyles, and directs attention to family-based interventions among this high-risk group as an urgent priority. The effectiveness of this approach is yet to be confirmed [30], although some studies have been successful in increasing youth physical activity by increasing family support [31-33].

Parental support can manifest in a variety of ways, through instrumental and affective mediation [34]. Cost, safety and access to facilities have been shown to limit physical activity among low SEP youth [35]. Instrumental support of low SEP families might occur through government-funded subsidies for associated costs, such as registration fees and sports uniforms, and the provision of more comprehensive public transport options to improve access to venues. Enhancement of emotional support mechanisms for physical activity participation, especially among young females in low SEP circumstances, is uniquely challenging. In particular, strategies to elevate girls' physical activity as a priority can be seen as incongruous with Australian sporting culture, which continues to marginalise female sport across all levels of competition [36].

Given the difficulties associated with changing family attitudes and practices [30], schools have the potential to address disparities in access to physical activity opportunities [37]. Nevertheless, Australian schools are challenged to meet the demands for instruction time from all learning areas. Regular physical activity as part of the curriculum is also threatened by ageing staff profiles and lack of expertise to conduct effective physical education (PE) and sport programs [38]. In light of a recent report of fewer physical activity opportunities in low SEP Australian schools [12], support for low SEP schools to appoint PE specialists would seem warranted, while more flexible timetabling could be adopted to free time for intra- and inter-school sport.

The YPAP construct 'is it worth it?' is comprised of benefits that relate to health and socialisation outcomes as well as enjoyment. Humbert and colleagues found that Canadian youth from both high and low SEP schools expressed the overwhelming importance of enjoyment as an attractant to physical activity [35]. Strategies that maximise enjoyment among participants are likely to be characterised by self-initiated behaviours and high levels of perceived choice [39]. According to the results of the current study, it is likely that these are important elements of any successful intervention, regardless of SEP.

Strengths and limitations of this study must be acknowledged. The sampling method ensured that schools in neighbourhoods towards the extremes of SEP were included. While response rates for the parent survey substantially reduced the final sample, there were no differences in mother education between those who did, and did not, return parent surveys. A parent survey was used to collect data on aspects of parent education and environmental barriers that are likely to be difficult for children to report. Self-reported physical activity has widely accepted limitations [40]. However, the use of the previous week as the sampling period and the absence of duration as a measured variable are positive features. Confining the sampling period to the previous week, and asking respondents to report frequency of activities during specified time periods, potentially reduce the effect of associative recall bias of self-reports [40] when compared to longer monitoring frames or when specific duration of physical activities are reported. Finally, as with all cross-sectional studies, causal relationships cannot be inferred from the observed correlations.

## Conclusion

Adolescence is a period of life consistently associated with declining physical activity levels, particularly among females [29,41,42]. Low SEP children often do not have the same access to convenient facilities for physical activity, compared to those from higher income families [16], and may be less likely to receive parental support [43]. Interventions to address inequities in physical activity opportunities need to assist low SEP parents to provide both instrumental and emotional support for their children. While acknowledging the limitation of the cross-sectional study design, the current study suggests that low SEP girls may be particularly dependent on social support to be physically active, underscoring the need to more specifically understand how parents stimulate girls' attraction to physical activity.

## Competing interests

The authors declare that they have no competing interests.

## Authors' contributions

JD acquired the funding, supervised the research, participated in the design of the study, performed the statistical analysis and drafted the manuscript. NL performed the research, collected the data, participated in the research design and coordination, and helped to draft and edit the manuscript.
